# The Multifunctional Family of Mammalian Fatty Acid–Binding Proteins

**DOI:** 10.1146/annurev-nutr-062220-112240

**Published:** 2023-05-19

**Authors:** Judith Storch, Betina Corsico

**Affiliations:** 1 Department of Nutritional Sciences and Rutgers Center for Lipid Research, Rutgers University, New Brunswick, New Jersey, United States; 2 Instituto de Investigaciones Bioquímicas de La Plata, CONICET-UNLP, Facultad de Ciencias Médicas, La Plata, Argentina

**Keywords:** lipid-binding proteins, fatty acids, lipid metabolism, lipid transport, metabolic diseases, cancer

## Abstract

Fatty acid–binding proteins (FABPs) are small lipid-binding proteins abundantly expressed in tissues that are highly active in fatty acid (FA) metabolism. Ten mammalian FABPs have been identified, with tissue-specific expression patterns and highly conserved tertiary structures. FABPs were initially studied as intracellular FA transport proteins. Further investigation has demonstrated their participation in lipid metabolism, both directly and via regulation of gene expression, and in signaling within their cells of expression. There is also evidence that they may be secreted and have functional impact via the circulation. It has also been shown that the FABP ligand binding repertoire extends beyond long-chain FAs and that their functional properties also involve participation in systemic metabolism. This article reviews the present understanding of FABP functions and their apparent roles in disease, particularly metabolic and inflammation-related disorders and cancers.

## INTRODUCTION

The relative insolubility of fatty acids and their potential toxicity in free form led to the search for intracellular noncatalytic binding proteins. The first fatty acid–binding protein (FABP) was detected in the cytosol of intestinal mucosa ~50 years ago (133), and this was followed by the identification of similar proteins in many other organs. FABPs are a family of 14–15 kDa carriers of small hydrophobic molecules abundantly expressed in tissues that are highly active in fatty acid (FA) metabolism. Originally proposed to be involved in lipid fluxes, in metabolism, and, subsequently, in signaling within their cells of expression, their participation in systemic metabolism is now recognized. To date, 10 FABP protein-coding genes have been identified in the human genome (*FABP1*–*9* and *FABP12*). They were originally named for the tissue in which they were first identified or mostly predominate; however, since many of the FABPs are expressed in multiple tissues, they are presently designated by number: FABP1 (LFABP, liver), FABP2 (IFABP, intestinal), FABP3 (HFABP, muscle and heart), FABP4 (AFABP, adipocyte), FABP5 (EFABP, epidermal), FABP6 (ILBP, ileal), FABP7 (BFABP, brain), FABP8 (PMP2 or P2, myelin), FABP9 (TFABP, testis), and FABP12. Although originally described as intracellular proteins, they are also found outside the cell and have been proposed to function via the circulation as well as intracellularly.

Phylogenetic analysis demonstrates strong evolutionary conservation of the FABP family. FABPs are present in a broad spectrum of animal species, and it has been estimated that they evolved approximately 1,000 million years ago, after the divergence of animals from plants and fungi, by subsequent duplications of a single ancestral gene generating a large number of tissue-specific homologs. Chromosomal mapping has shown that several FABP members (*FABP4*, *−5*, *−8*, *−9*, and *−12*) colocalize at the same chromosome, providing further evidence of their evolutionary origin. Furthermore, the overall gene structure of FABPs is highly conserved, consisting of four exons separated by three introns (161).

The regulated expression of FABPs began to be appreciated immediately after their discovery, when it was observed that intestinal FABP concentration was increased in animals maintained on a high-fat diet (HFD) (134). The expression of these proteins is mostly regulated at the transcriptional level through interactions at conserved motifs within *FABP* gene promoters with transcription factors that either activate or repress their expression. *FABP* genes have several regulatory elements, differing in specificity and function, and are differentially regulated in different species, tissues, and cell types depending on the different repertoire of transcription factors and their interactions. Most of these regulatory elements direct tissue-specific expression or are associated with lipid processing. For instance, the presence of peroxisome proliferator response elements has been detected in *FABP1*, *FABP3*, *FABP4*, *FABP5*, and *FABP6* genes; the peroxisome proliferator activated receptors (PPARs) are all regulated via activation by FAs or other ligands that are targeted to PPARs by FABPs in a feedback loop for endogenous and exogenous ligands.

**Figure 1:**
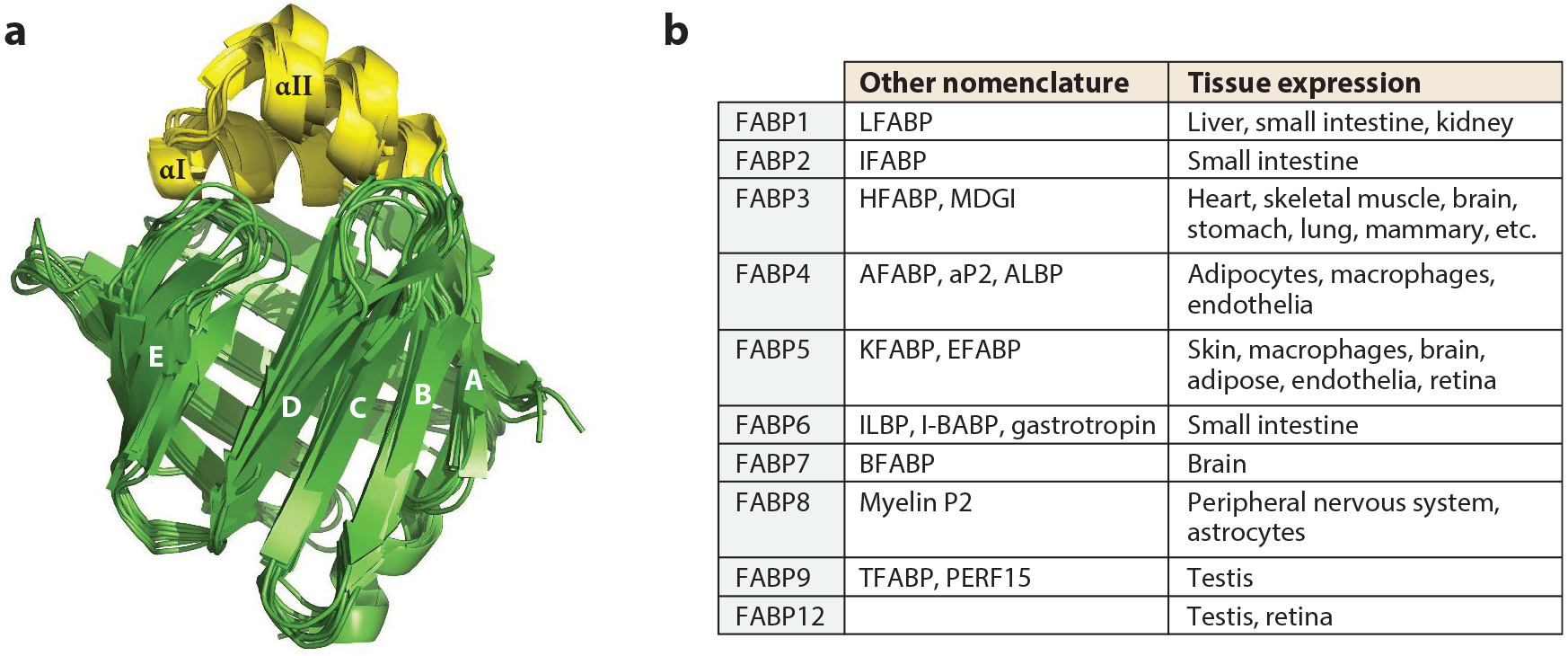
The family of mammalian fatty acid–binding proteins (FABPs). (*a*) Superimposition of crystal structures of unliganded FABP1–9 generated in PyMOL (FABP1, 2F73; FABP2, 1IFC; FABP3, 6AQ1; FABP4, 3RZY; FABP5, 4LKP; FABP6, 5L8I; FABP7, 1FDQ; FABP8, 3NR3; and FABP9, 4A60). Panel adapted from Reference 119 with permission from Elsevier. (*b*) Table listing the mammalian FABPs, whose numbers reflect the order of their initial discovery.

In spite of the variable sequence identity along their 126–134 amino acid length, ranging from 20% to 70%, all FABPs share a conserved tertiary structure consisting of a slightly elliptical beta barrel comprising two nearly orthogonal five-stranded β sheets that wrap around a solvent-accessible ligand-binding cavity capped by a small helix-turn-helix lid; structures are shown in Figure 1 along with a table of the 10 mammalian FABPs, numbered in order of chronological identification. While high-resolution structures obtained by X-ray crystallography show that unbound and ligand bound proteins have very similar structures, nuclear magnetic resonance (NMR) analyses demonstrate considerable differences in the distal half of the αII helix and the turn between β strands C and D; this flexibility is implicated in ligand entry and exit, and thus the structure comprising the helical domain and associated β-strand turns is called the portal region. These portal domain elements are more disordered in the unliganded state and show stabilization of the secondary structure by ligand binding, suggesting that during ligand exit/entry, the portal region undergoes a conformational change, which allows the FA to pass through the portal. Several studies combining NMR, mutagenesis, and molecular dynamics simulation have demonstrated that a transient local unfolding in the αII helix provides a temporary opening in the portal region for ligand association or egress (60, 185).

FABPs bind saturated and unsaturated long-chain fatty acids (LCFAs) with an affinity that increases with ligand hydrophobicity (165). Most bind one ligand molecule, except for FABP1 and −6, which present a 2:1 ligand:protein stoichiometry. FABP6 has a higher affinity for bile acids, and FABP1 as well as other family members are now shown to bind other hydrophobic molecules including lysophospholipids, fatty acyl coenzyme A, cholesterol, bile acids, heme, and monoacylglycerols. In the last decade, several studies have demonstrated that FABPs can also bind and transport endocannabinoids, cannabinoid-like molecules, and phytocannabinoids, thus expanding the spectrum of FABP functions to include endocannabinoid signaling processes (34, 80, 97). FABPs bind several classes of lipophilic drugs, and FABP1 and FABP2, among other FABPs, were found to bind hypolipidemic fibrates, with differential binding specificities and consequent PPAR activation (72). Various small molecules that can regulate the function of FABPs have been developed in recent years, with initial inhibitors targeted at FABP4 (46). Derivatives of these compounds or other small molecules have been developed that specifically bind to FABP3, FABP5, and FABP7 (10, 155).

Due to their binding properties and intracellular localization, FABPs were initially proposed to function as intracellular fatty acid transport proteins. This function has been supported by cell-based assays and gene knockout (KO) studies, which show a positive correlation between FA uptake and FABP levels. Their apparent action as regulators of FA uptake may depend on their interaction with plasma membrane FA transport proteins, such as CD36, or with membrane lipids after transmembrane fatty acid translocation (“flip-flop”). Once inside the cell, FABPs may target their ligands to specific subcellular destinations. The analysis of protein-membrane interactions based on in vitro kinetics studies has resulted in the categorization of FABPs in two classes, those that exchange FA through direct collisional interaction of the FABP with a target membrane and those that employ an aqueous diffusion-based mechanism. The helix-turn-helix portal domain appears to be the key structural determinant of the FA transfer mechanism for a particular FABP (166). Interestingly, the interaction between FABP8 (also known as myelin P2) and two membrane surfaces has been demonstrated, with membrane interactions at the portal domain as well as a second region on the opposite side of the protein; these interactions are thought to be important for peripheral nerve myelin sheath structure and function (148).

As detailed below, the functional properties of the FABPs have defied a singular paradigm. While initially proposed as the intracellular counterpart of serum albumin, serving to bind and transport FA inside their cell of expression, decades of research have shown not only that their ligand binding repertoire extends beyond LCFA but also that their functional properties extend beyond an intracellular lipid-binding role. The fact that multiple functions are being reported for the FABPs is perhaps not surprising, in that 10 separate protein-coding genes have been identified in mammals, and many tissues express not only one but two and sometimes more FABPs. Such genetic diversification implies functional diversity, and this indeed is borne out by research, done largely in rodent models, which points to a plethora of functional roles for specific members of the FABP family. For example, the dysregulation of FABP expression and function has been observed in numerous inflammatory diseases including obesity and a range of obesity-related disorders (Table 1). The association of FABPs with multiple types of cancer is increasingly appreciated and actively investigated. Examples are provided in Table 2 and extensively reviewed by McKillop et al. (121). Typically, the functions of the FABPs appear to be dependent on the lipid-binding properties of the protein, but exceptions have been reported.

Given the expanding understanding of FABP ligand-binding properties and the increased appreciation of different functions and tissue-specific roles for specific members of the family, this review attempts to summarize key research findings leading to our current state of knowledge of the roles played by each member of the FABP family in mammalian health and disease.

## FABP1

FABP1 is abundantly expressed in hepatocyte cytosol (2–10% of the total cytosolic protein), small intestinal enterocytes, and, to a lesser extent, renal proximal tubular cells. Along the anterior-posterior axis of the small intestine, FABP1 is expressed in a gradient with a maximum at the jejunum, the site of maximal lipid absorption (165).

FABP1 is unusual in its binding properties, possessing a larger binding cavity, relative to other family members, that accommodates two ligand FA molecules. NMR-derived structures reveal pronounced backbone flexibility in the residues around the portal region in both the apo- and the ligand-bound protein, which seem to be essential to enable ligand binding. FABP1 was also considered atypical until recently for binding many bulky and rigid ligands such as lysophospholipids, bile salts, monoacylglycerols, and other hydrophobic ligands. The NMR solution structure of FABP1 with bound monoolein resembles that of the FA-liganded protein, with two bound ligands, while bile salts bind with a 1:1 stoichiometry (41). Like other members of the FABP family, FABP1 is involved in the intracellular transport of endocannabinoids (ECs) and related molecules (97). FABP1 is the predominant EC-binding protein in the liver and is proposed to be involved in the cellular uptake and transport of ECs to intracellular degradation pathways (120). FABP1 also binds exogenous phytocannabinoids including Δ9-tetrahydrocannabinol (THC), the main psychotropic component of cannabis, with 1:1 stoichiometry (34).

There is compelling evidence that FABP1 directly interacts with PPARα and is involved in shuttling activating ligands from the cytosol to the nucleus to regulate lipid metabolism, differentiation, inflammation, and cell survival. The mechanisms of FABP1 access to the nucleus and PPAR activation by specific ligands have been studied in detail, and these processes may provide a means to pharmacologically modulate PPARs, which play a role in the development of obesity, cardiovascular diseases, type 2 diabetes (T2D), and atherosclerosis (142).

After absorption in the small intestine, lipids are either packaged into chylomicrons or stored as cytosolic lipid droplets (CLDs). FABP1 is associated with CLDs in the jejunum where it has been shown to modulate triglyceride hydrolysis (67). FABP1 also participates in lipoprotein biogenies in the intestine, as part of a multiprotein complex that selects prechylomicrons as cargo and buds transport vesicles from the endoplasmic reticulum (ER). FABP1 is released from the complex after Sar1b, one of the members of the complex, is phosphorylated by protein kinase C zeta (PKC-ζ), whereupon FABP1 binds to the ER and organizes the prechylomicron transport vesicle budding complex (159). Employing the Caco-2 cell model of human intestinal epithelial cells, it was demonstrated that cells with decreased expression of FABP1 show a decrease in FA uptake rate and reduced basolateral FA secretion. The distribution of secreted lipid shows an increase in FA/triacylglycerol ratio compared with that of control cells, probably due to the role of FABP1 in chylomicron assembly (146). Interestingly, intestine-specific disruption of PPARα and FABP1 in mice (196) suggests that intestinal PPARα signaling promotes nonalcoholic steatohepatitis (NASH) via the modulation of intestinal FABP1, which regulates dietary FA uptake, highlighting the potential importance of intestinal PPARα and FABP1 as targets for the treatment of NASH.

A direct comparison of FABP1 and FABP2 null animals revealed highly divergent phenotypes in response to an HFD (49). The FABP1^−/−^ mice displayed increases in body weight and food intake, relative to wild-type (WT) mice. In contrast, FABP2^−/−^ mice under identical conditions displayed less weight gain compared with that of WT mice, accompanied by a reduction in food intake. Fuel utilization was also different, with FABP1^−/−^ mice preferentially utilizing lipids and FABP2^−/−^ mice preferentially metabolizing carbohydrates. Notably, despite their increased body weight, the FABP1^−/−^ mice had normal glucose and insulin levels and increased spontaneous activity compared with those of WT mice. Additionally, FABP1 ablation was accompanied by major metabolic alterations in skeletal muscle, including higher energy substrate levels and mitochondrial respiratory capacity, and increased oxidation of circulating FAs during exercise, all of which protect FABP1 KO mice from the decline in exercise performance that typically accompanies high-fat feeding. Thus, FABP1^−/−^ mice appear to present a model of metabolically healthy obesity (188).

Within the intestine itself, mucosal lipid metabolism was differentially modified, with significant decreases in FA incorporation into triacylglycerol (TG) relative to phospholipid (PL) in FABP2^−/−^ mice, whereas FABP1^−/−^ mice displayed reduced monoacylglycerol incorporation in TG relative to PL, as well as reduced FA oxidation, possibly indicating participation of FABP1 in FA trafficking to oxidative pathways and explaining, at least in part, the obese phenotype. FABP1^−/−^ mice on an HFD had longer intestinal transit time, less fecal output, and more guilds containing bacteria associated with obesity (182). Higher mucosal levels of the orexigenic endocannabinoids arachidonoylethanolamine (AEA) and 2-arachidonoylglyccerol (2-AG) were also observed in FABP1 KO mice, a finding that may be related to the increased appetite in these mice (49). By contrast, FABP2 KO mice showed decreased 2-AG levels in the intestinal mucosa, perhaps explaining the observed decrease in food intake.

Interestingly, *FABP1* gene ablation in male mice significantly increased brain levels of EC (116), presumably associated with increased availability of serum FA for uptake by brain, although this was not directly demonstrated. As noted earlier, FABP1 binds exogenous phytocannabinoids such as THC. Primary hepatocytes from FABP1 KO mice showed a reduced production of THC metabolites compared with that of WT cells, suggesting a defect in intracellular THC transport to degradative enzymes. Moreover, administration of THC to FABP1 KO mice potentiated the physiological and behavioral effects of THC (34). These studies highlight the peripheral EC system as a possible target for safe antiobesity agents and substance use disorder treatments.

In the liver, FABP1 is predominantly expressed in hepatocytes but is also found in hepatic stellate cells (HSCs), which are the major fibrogenic effector cell type when activated. FABP1 deletion in hepatocytes attenuates both diet-induced hepatic steatosis and fibrogenesis, characteristics of NASH, whereas HSC-specific FABP1 deletion did not alter the fibrogenesis (130). Further indication that FABP1 may be involved in the development of nonalcoholic fatty liver disease and NASH comes from a study showing that activation of the glucagon-like peptide-1 receptor reduces oleate-induced steatosis in HepG2 cells by reducing FA uptake and transport via FABP1 downregulation (86). A role for FABP1 in metabolic disease is also suggested, albeit indirectly, by the presence of a single-nucleotide polymorphism (SNP) in the coding region of human FABP1 that results in a threonine to alanine replacement (T94A). This SNP is one of the most prevalent polymorphisms in the FABP family in the human population and is associated with serum lipid abnormalities (178).

Altogether, it is increasingly clear that FABP1 not only is involved in intracellular lipid trafficking and metabolism but also has profound systemic effects, which may arise via alterations in signaling pathways that regulate activity, food intake, and energy utilization. Indeed, the systemic effects may be of greater impact than intracellular function, at least in the intestine, as an FABP1/FABP2 double KO mouse integrated the whole-body phenotypes of the two individual KO strains but was not found to have dietary fat malabsorption. These results imply that while the intestinal FABPs have important effects on systemic energy homeostasis and are involved in dietary lipid processing, they are not essential for net intestinal lipid absorption (48).

The expression of FABP1 is associated with different types of cancer. FABP1 is highly expressed in hepatocellular carcinoma and is proposed to promote angiogenesis, tumorigenesis, and metastasis through VEGFR2/SRC proto-oncogene tyrosine-protein kinase signaling and the focal adhesion kinase/cell division cycle 42 pathway (93). In a small intestinal adenoma mouse model, FABP1 deletion protects against tumorigenesis and induces a change in the intestinal mucosa FA profile accompanied by changes in expression of enzymes involved in FA elongation and desaturation (32). In line with this, it was shown using small intestine epithelial cells that the RNA-binding protein DDX5 posttranscriptionally enhances FABP1 expression in intestinal cells, promoting tumorigenesis (1).

## FABP2

FABP2 is expressed in human small intestine with highest concentrations in jejunum. It is coexpressed with FABP1 in fully differentiated enterocytes, and their mRNAs are the most abundant translatable RNA sequences in the gut epithelium. Their intracellular localization is modified after fat feeding from the apical pole to the entire cytoplasm, supporting a role in trafficking of diet-derived lipids (15, 165).

The human *FABP2* gene is regulated by HNF4α, HNF1α, and GATA transcription factors during development and intestinal differentiation. *FABP2* gene downregulation in HNF4 KO mice suggests that FABP2 is part of the metabolic infrastructure that carries out fatty acid oxidation necessary for intestinal stem cell renewal in the crypts of intestinal epithelia (21). FABP2 expression in intestinal cells is also regulated through the Wnt/β-catenin/PPARγ signaling pathway, which participates in development and renewal of the intestinal epithelium (89). It is important to note that dysregulation of this process is related to several pathological disorders including colorectal cancer and inflammatory bowel disease. Taken together, these characteristics suggest the participation of FABP2 in intestinal development and differentiation in addition to a role in dietary lipid processing.

FABP2 binds FAs in a 1:1 stoichiometric ratio with high affinity. It has been shown that human FABP2 also binds a range of different lipophilic drugs at two different sites and via different modes of interaction (141). Binding at the FA site appears to be driven by polar interactions with residues at the bottom of the FABP2-binding cavity. Many of the drugs that bind FABP contain carboxylate functional groups, suggesting that they may bind in a manner similar to that of FA. However, the presence of a second binding site in FABP2, which is able to bind ligands adjacent to the portal site, raises the possibility that the binding repertoire of FABP2 may be broader than previously recognized (141). FABP2, similar to FABP1, binds hypolipidemic drugs and promotes the activation of PPARα; nevertheless, they both differentially modulate PPARα activation in a ligand selective manner (72). FABP2 also binds endocannabinoids, but with a different affinity compared with that of FABP1 (97). As a whole, these studies are suggestive of different roles for FABP1 and FABP2 in the intestine and open the possibility for the development of targeted delivery of drugs in different tissues.

Animal studies also strongly support different roles for FABP2 and FABP1 in the intestine. As mentioned above, FABP2 KO mice on an HFD displayed reduced weight gain and fat mass, opposite to the phenotype of the FABP1 KO mouse. The lower body weight was accompanied by a reduction in food intake and decreased 2-AG levels in the intestinal mucosa (49). It was further shown that FABP2^−/−^ mice have more rapid intestinal transit, increased fecal excretion, and increased intestinal motility, likely related to the decrease in 2-AG levels, as well as increased fragility. This suggests that FABP2 null mice are malabsorbing nutrients in general, which likely contributes to their leaner phenotype (96). In line with this, gut microbiota of FABP2^−/−^ mice showed an increase in antiobesity-promoting guilds after HFD feeding relative to WT mice (182). The ablation of FABP2 induces differential alterations in male and female mice. Analysis of the small intestine transcriptome shows that the ablation of the FABP2 gene causes a much larger degree of metabolic disturbance in male than female FABP2^−/−^ mice; the major gene networks shown to be differentially expressed include transcriptional signaling processes (23). Overall, the studies in genetically modified animals including the single KO and the FABP2/FABP1 double KO mouse (48) suggest that like FABP1, FABP2 may participate in lipid sensing and signaling in the small intestine with subsequent effects on whole-body energy homeostasis.

A polymorphism at codon 54 of FABP2 that results in the substitution of a Thr for an Ala is associated with higher fat oxidation and insulin resistance in Pima Indians, a group with the highest reported prevalence of T2D. Epidemiological studies in other ethnic groups have demonstrated association of the T54A polymorphism with postprandial hypertriglyceridemia (51) as well as central obesity and metabolic syndrome (64).

With the aim of evaluating effects of the cancer cell microenvironment, the presence of different FABPs was analyzed in a cell line derived from a patient with breast adenocarcinoma. Only FABP2 and FABP3 mRNAs were found in the breast cancer cells; FABP2 expression was found to be modulated by hormones and after exposure to media conditioned by fibroblasts with increased lipid levels (113).

## FABP3

FABP3 constitutes 4–8% of the cytosolic protein in mammalian heart and is highly expressed in skeletal muscle and, to a lesser extent, in stomach, brain, lung, mammary gland, placenta, and immune cells. It binds both saturated and unsaturated LCFAs, showing a higher affinity for *n*–6 FA. It also binds oxygenated fatty eicosanoids derived from arachidonic acid, which regulate vascular tone and blood flow (162). Similar to FABP5 and FABP7, which are also expressed in the brain, FABP3 binds intracellular endocannabinoids, although with lower affinity (80).

In the FABP3-deficient mouse, a change in fuel utilization from LCFA to glucose, accompanied by decreased exercise tolerance and cardiac hypertrophy, were observed (11, 37, 149). The increase in cellular TG levels in skeletal muscle and heart that is known to occur under a chronic HFD was prevented in the FABP3 KO mice (37). In a mouse model for myocardial infarction (MI), it was demonstrated that ischemic conditions increased FABP3 expression under the regulation of hypoxia-inducible factor (HIF)-1α. Overexpression of FABP3 after MI induced myocyte apoptosis and aggravated cardiac dysfunction. Conversely, deficiency of FABP3 exerted protective effects against ischemic heart injuries. It was also observed that FABP3 upregulated MAPK phosphorylation and decreased phosphorylated Akt levels, which may account for the augmentation of apoptosis and remodeling after MI. FABP3 was therefore suggested as a potential therapeutic target to treat ischemic and related heart diseases (202); however, the above-mentioned effects of FABP3 inhibition on fuel selection and cardiac hypertrophy must be considered.

In the brain, FABP3, −5, and −7 have distinct spatiotemporal expression profiles that correlate with specific developmental stages and processes. FABP3 mRNA is barely detectable during embryonic development of mouse brain, and the levels gradually increase after birth until adulthood, suggesting its association with maintenance of the differentiation status of adult brain cells. Elevated levels of FABP3 in cerebrospinal fluid and in serum of Parkinson’s disease (PD) patients have been associated with motor and cognitive decline and neurodegeneration-specific biomarkers (102). In mice lacking the *FABP3* gene who were subjected to treatment to induce PD-like symptoms, midbrain cells lacked the characteristic accumulation of α-synuclein (αSyn) typically observed in WT mice, and the mice were more resistant to neurodegeneration and motor deficits (156). It was demonstrated that FABP3 interacts directly with αSyn and enhances αSyn oligomerization in purified systems and cell culture. The interaction with FABP3 involves the C-terminal region of αSyn, and a synthetic peptide corresponding to this region is capable of disrupting the αSyn–FABP3 complex; thus, it was suggested that administration of this peptide might be useful in preventing the progression of PD (45). Using primary cultured neurons from FABP3 KO mice, it was further demonstrated that FABP3 is critical for αSyn uptake into dopaminergic neurons via caveolae-mediated endocytosis coupled with dopamine D2L receptors, suggesting a novel pathogenic mechanism for synucleinopathies (85). Altogether, FABP3 appears to be involved in αSyn uptake into dopaminergic neurons and to enhance the propagation of αSyn aggregates, suggesting FABP3 as a potential therapeutic target for synucleinopathies.

FABP3 is expressed in human placental third trimester trophoblasts. As was found in the heart, placenta from FABP3 KO mice showed a decrease in polyunsaturated fatty acid (PUFA) transport while glucose transport was increased, suggesting metabolic reprogramming and highlighting the role of FABP3 in fetal development (75).

FABP3 may also participate in regulating immune cell differentiation. B cells from FABP3 KO mice exhibited significantly reduced immunoglobulin M (IgM) secretion, and it is proposed that FABP3 regulates the generation of IgM-producing cells by controlling histone acetylation of the promoter region of a transcription factor that regulates B cell differentiation into antibody-secreting cells (90).

Participation of FABP3 in cancer progression has been reported in different types of tumors, albeit with conflicting effects. In human non-small cell lung cancer (NSCLC) tissue, increased expression of FABP3 was associated with advanced tumor metastasis and shorter overall survival (171). According to the Cancer Genome Atlas database, FABP3 mRNA expression is downregulated in breast cancer tumor tissues. Nevertheless, patients with high FABP3 protein expression in both breast tumor tissues and the tumor adipose microenvironment had worse prognosis (110). Moreover, as found for FABP2, FABP3 mRNA was discovered in breast cancer culture cells where its expression is modulated by hormones and after exposure to media conditioned by fibroblasts (113). FABP3 protein expression is correlated with a poor prognosis in patients with gastrointestinal stromal tumors and esophageal cancer (22, 29). Overall, FABP3 participation in tumor progression is uncertain mainly due to the fact that FABP3 may be expressed in tumor tissue as well as in nontumor tissue such as immune cells and stromal cells, and its role may be context specific in malignant progression.

## FABP4

FABP4, also termed AFABP and aP2, is abundantly expressed in white and brown adipocytes as well as macrophages. Endothelial cell expression of FABP4 has also been repeatedly demonstrated. The functions of FABP4 within these cells are thought to be related to its lipid-binding and transport properties and to its participation in the regulation of gene expression. Although not routinely examined in studies of FABP4 action, ligand dependence is generally observed when this question is directly interrogated (181, 190). Additionally, ligand-dependent entry into the nucleus via a noncanonical nuclear localization signal has been reported (131), and molecular dynamics simulations support the interaction of linoleate-FABP4 with the α-karyopherin protein of the nuclear importin complex (4).

Within the adipocyte, FABP4 appears to play a stimulatory role in lipolysis via protein-protein interaction with hormone-sensitive lipase (HSL). While FABP4 might logically be considered to enhance lipolysis by serving as a sink for the FAs released by HSL, it was found that stimulation of lipolytic activity occurred only for holoFABP4 (165). An interesting functional interaction with another key TG lipase in adipose tissue, ATGL (adipose triacylglycerol lipase), via direct binding of FABP4 to the ATGL activator Cgi-58 (comparative gene identification-58), has also been shown; the FABP4–Cgi-58 interaction is stimulatory for ATGL lipolytic activity (67). FABP4 actions in adipocytes and macrophages may also be related to its activation of PPARγ activity via nuclear translocation (131), although suppression of PPARγ via increased proteasomal degradation has also been reported (53). In brown adipocytes, FABP4 was found to indirectly stimulate adaptive thermogenesis by reducing LXRα, which results in induction of type 2 iodothyronine-deiodinase conversion of T4 to the active hormone, T3 (158).

In macrophages, FABP4 has been shown to support an inflammatory M1 state via binding and preventing the hydrolysis of leukotriene A4 and by upregulating expression of the leukotriene B4 receptor BLT1R, thereby increasing LTB4-mediated signaling (66). Macrophage FABP4 has also been reported to inhibit autophagy by inhibiting JAK2 activity and Atg7 expression, thus promoting ER stress in response to inflammatory stimuli such as palmitic acid treatment (69). Further, macrophages incubated with saturated fatty acids were found to produce cytotoxic ceramide species in an FABP4-dependent manner, implicating delivery of saturated fatty acid substrate to ceramide synthases (200). A role in inflammation for macrophage FABP4 is also suggested by its downregulation of uncoupling protein 2 (UCP2) and SIRT3 levels as well as reduced cellular redox capacity, with FABP4 ablation correlated with reductions in the unfolded protein response and release of inflammatory cytokines (163, 189, 190). Interestingly, and in contrast to its proinflammatory actions in the macrophage, an antioxidant and anti-inflammatory role for FABP4 in the adipocyte was suggested by studies in the 3T3L1 adipocyte cell line where silencing of FABP4 led to increased sensitivity to hydrogen peroxide and increased ER stress (83).

In addition to adipocytes and macrophages, FABP4 is expressed in endothelial cells in response to Notch, which was shown to bind to the FABP4 promoter, and secondary to vascular endothelial growth factor A (VEGFA), with downstream effects of VEGFA on angiogenesis dependent on FABP4 expression (65). A role for endothelial FABP4 in angiogenesis via regulation of the expression of stem cell factor c-kit was also suggested by studies in human umbilical vein endothelial cells (33). FABP4 and FABP5 in muscle capillary endothelial cells were proposed to regulate FA uptake by brown adipose tissue (BAT), with dual KO leading to reductions in BAT thermogenesis as well as reduced availability of triacylglycerol in BAT and glycogen in muscle (170). In double KO mice, the absence of the FABPs in heart and muscle capillary endothelial cells was suggested to be responsible for the reduced uptake of FA and compensatory increase in glucose uptake observed in the heart (76).

Perhaps the most intriguing FABP4 functions arise secondary to its proposed actions as a protein secreted by adipose tissue into the circulation (16). It has been repeatedly shown that circulating levels of FABP4 are related to important aspects of metabolic physiology and, in general, higher levels of plasma FABP4 are reported in obesity and inflammatory states. Addition of FABP4 to isolated pancreatic islets was shown to increase insulin secretion (183), and chronic administration of FABP4 in mice led to an insulin-resistant state (92). Incubation of HepG2 cells with FABP4 also increased markers of insulin resistance, as well as ER stress and apoptosis (14). In human carotid artery smooth muscle cells, FABP4 treatment led to increased cell proliferation and migration (58). Circulating FABP4, which as noted is elevated in obesity, is considered an important factor promoting vascular inflammation and plaque deposition in the development of atherosclerosis (47, 99). FABP4 inhibitors are generally shown to be protective against these metabolic and inflammatory effects, supporting an etiologic role for FABP4 in the development of numerous obesity-related disorders.

Thus, FABP4 has been variously termed an adipokine and more recently a hormone, in that circulating FABP4 expressed in adipose tissue influences downstream tissues where it is not expressed, such as the pancreas, muscle, and numerous other target tissues.

It is worth noting that the basic mechanisms underlying circulating FABP4 actions, from its secretion to its function in the circulation, appear to be atypical in nature. FABP4 does not contain a canonical secretory signal in its primary amino acid sequence. It was reported to be secreted in extracellular vesicles in response to lipolytic stimuli (38, 125, 183); secretion was found to be calcium dependent and to occur via a membrane-bound vesicular compartment involving the endolysosomal system but not the typical ER to Golgi protein secretory pathway (150, 179). The involvement of autophagic membrane compartments has been found by some but not others (77, 179), and a dependence on sirtuin-1 signaling has also been observed (77). Limited information about potential mechanisms of FABP4 uptake by distal tissues and cells has thus far been reported. Endothelial cell uptake was shown to be dependent on a specific domain of the plasma membrane-bound protein cytokeratin 1 (118), while in the kidney circulating FABP4 is filtered in the glomerulus but reabsorbed via binding to megalin in proximal tubule endothelial cells (157). Interestingly, FABP4 may also regulate distal tissue function by acting in the blood-stream rather than solely via its uptake and action within target tissues. A complex between FABP4 and two circulating nucleoside kinases, adenosine kinase and nucleoside diphosphate kinase, appears to regulate the activities of the kinases, leading to an increase in the ATP to ADP ratio, which was shown to be associated with a purinergic receptor P2Y-mediated increase in glucose-stimulated insulin secretion from pancreatic β cells. Perhaps surprisingly, these actions appear independent of FA binding by FABP4 (144). Thus, the role of FABP4 in modulating glucose homeostasis may occur through this unusual mechanism of altering extracellular metabolite fluxes and without a typical receptor for either uptake or for signal transduction at the cell surface. It remains to be seen whether the relationships between circulating FABP4 and obesity, coronary heart disease, and various types of cancer are also occurring through similar indirect extracellular mechanisms.

FABP4 has been reported to be involved in numerous types of cancer; high expression has been shown in colon cancer tissue (173), liver cancer stem cells (193), glioblastoma tissue and cell lines (111), ovarian cancer (56) and acute myeloid leukemia (195), among others. Exogenous FABP4, delivered either directly or by coculture with adipocytes, has also been demonstrated to support cancer cell proliferation and migration (88, 127, 181). Elevated FABP4 has been observed in tumor-associated macrophages in neuroblastoma (123) and hepatic stellate cells in hepatocellular carcinoma associated with metabolic syndrome (26). As with metabolic syndrome–related disorders, numerous studies of a variety of cancers, in cultured cells as well as mouse models, have shown that pharmacologic or genetic inhibition of FABP4 diminishes cell proliferation, migration, and invasiveness (71, 127, 193).

While FABP4 levels outside of the adipocyte and macrophage and perhaps endothelial cells are generally considered to be low in the normal state, high levels of expression have been widely reported in various tissues not only in cancers but also in other types of pathological states. These include increased levels in kidney tubular epithelia in kidney fibrosis (24), in septic heart muscle (167), and in retinal tissue in diabetic retinopathy (39). In these conditions, too, pharmacologic or genetic suppression of FABP4 is shown to be protective.

Thus, it would seem that inhibition of FABP4 function may be therapeutic for numerous prevalent diseases including diabetes, cancer, and atherosclerosis, and numerous small molecule inhibitors have been developed (44). As yet, however, there have been no clinical trials for FABP4 (or other FABP) inhibitors. If the protein is indeed functioning in the circulation itself or via the circulation, systemic administration rather than targeted delivery might be expected to affect many tissues. Some caution also arises from reports that FABP4 null mice were shown to exhibit autism-like behaviors relative to their controls, and that FABP4 levels are lower in children with autism (115). It was also shown that FABP4 in lung macrophages is important for neutrophil recruitment and that FABP4 null mice were less resistant to bacterial pneumonia (103). Nevertheless, the promise of regulating FABP4 function to prevent or treat chronic disease is appealing. Future work will need to address questions of specificity—in some studies, other FABPs have similar effects (98); if FABP4 is secreted within exosomes then hundreds of other proteins are secreted alongside it. The importance of FA binding, and indeed the roles of specific lipid ligands, needs further exploration. Additionally, if secretion is occurring via extracellular vesicles, the molecular mechanism by which FABP4 is accessed by other circulating proteins that may be functional partners needs to be understood.

## FABP5

Unlike most other FABPs, which have more or less restricted tissue expression patterns, FABP5 (along with FABP3) is expressed in a relatively large number of tissues. Originally identified in skin and labeled as KFABP or EFABP (for keratinocyte or epidermal FABP), it is also expressed in macrophages, endothelial cells, the gastrointestinal tract, the brain, and, at low levels, adipocytes.

Under normal physiological conditions, FABP5 appears to have numerous tissue-specific functions, sometimes playing opposing roles in different tissues. In lung macrophages, for example, FABP5 has been shown to promote anti-inflammatory processes and be protective against infection (36, 91), and it was found that posttranslational *S*-glutathionylation of FABP5 increases its translocation to the nucleus where it stimulates an anti-inflammatory gene expression profile (61). In liver macrophages, on the other hand, increased anti-inflammatory cytokines were found upon FABP5 knockdown, possibly via accumulation of oleate and inhibition of the nuclear factor kappa B (NF-κB) pathway (70, 126). Regulation of inflammation by FABP5 in macrophages appears to involve its role in the induction of prostaglandin E2 synthesis via upregulation of the prostaglandin E synthetase 1 enzyme, which in turn is upregulated by NF-κB binding to the PGES-1 promoter (13). Its role in the skin appears to involve normal keratinocyte differentiation via uptake of linoleic acid and its subsequent conversion to the eicosanoid 13-hydroxyoctadecadienoic acid [13(S)-HODE], which in turn increases the expression of keratin 1 (135). Endothelial cell FABP5 was found to promote docosahexaenoic acid (DHA) and other fatty acid uptake across the blood–brain barrier (BBB) (100, 139), and the permeability of the BBB to immune cell infiltration was also reported to be regulated by FABP5 via protein-protein interaction with calnexin (140). A double KO of FABP5 and FABP4 was shown to result in decreased fatty acid uptake into muscle endothelial cells and BAT, thereby not only regulating thermogenesis and energy homeostasis but also resulting in higher plasma FA levels and increased liver TG accumulation (169, 170). In a human retinal pigment epithelial cell line, knockdown of FABP5 led to decreased secretion of apoB100, resulting in its accumulation as well as the buildup of intracellular TG (184). Interestingly, in a manner similar to the regulation of lipoprotein secretion by FABP1 (159), FABP5 is reported to regulate ER to Golgi trafficking of large coat protein complex II (COPII)-coated vesicles by binding to the vesicle trafficking protein complex and modulating the GTPase activity of SAR1, as shown using cell-free vesicle trafficking analysis with subcellular components from a hepatoma cell line (122). A role in fat-stimulated but not glucose-stimulated secretion of the hormone GIP from enteroendocrine K cells has also been shown (154).

FABP5 binding of AEA and 2-AG plays an important role in its apparent actions in a wide variety of EC system–based physiological functions including pain, memory, fear and anxiety, and inflammation (12). FABP5 is involved in regulating AEA and 2-AG levels via intracellular EC transport to the inactivating enzymes FAAH and MGL (78), participates in EC signaling at both excitatory and inhibitory brain synapses (40), and is involved in retrograde endocannabinoid transport at central glutamate synapses (63).

The role of FABP5 in such a wide range of intracellular functions may be secondary, at least in part, to its ligand-dependent translocation to the nucleus and ligand transfer to PPAR transcription factors, as proposed by Noy (131). Such ligand binding and transport-dependent modulation of gene transcription, which in turn could control the numerous metabolic pathways and signal transduction cascades associated with FABP5 actions, may underlie many of its reported functions, as noted earlier for FABP4. Interestingly, differential effects of saturated versus unsaturated fatty acids, as well as differences between unsaturated fatty acid molecular species, may arise secondary to ligand-dependent interactions of FABP5 with different transcription factors, as well as the relative amounts of FABPs and retinoid-binding proteins, and of PPAR and RAR transcription factors (131).

FABP5 plays important roles in the development of several chronic diseases including cardiovascular disease where, as found for FABP4, macrophage-specific deletion of FABP5 was protective against development of atherosclerosis (7). It was also reported, however, that FABP5-deficient mice were susceptible to increased cardiac injury secondary to reduced cardiac mitochondrial function (50). Most extensively examined has been the role of FABP5 in numerous types of cancer (186). In virtually all cancers studied, including hepatocellular carcinoma, gastric and colon cancers, glioma, skin cancer, lung cancer, renal cancer, prostate cancer (PC), and breast cancer, FABP5 expression is associated with increased tumor growth, migration, and/or invasiveness. Thus, in many cell-based and in vivo studies, FABP5 deletion, knockdown, or inhibition has been shown to reduce cell proliferation, including in PC (132), glioma (180), colorectal cancer (153), gastric cancer (201), lung adenocarcinoma (52), and renal clear cell carcinoma (114). Reported mechanisms underlying these effects are quite varied and include protein-protein interaction and modulation of HIF-1α (153) or estrogen-related receptor (ERR)-α (152), regulation of TNF-α-dependent NF-κB signaling (180) or PI3 kinase/Akt signaling (114), and regulation of lipid metabolism (18, 52) or of angiogenesis via VEGFA-dependent signaling (138). It is noteworthy, however, that several reports indicate that inhibition/knockdown of FABP5 could have an opposite effect, resulting in more skin tumors (199) and increased lung cancer metastases (194).

## FABP6

FABP6 (also known as ILBP, I-BABP, or gastrotropin) has high affinity for bile acids (BAs). Like FABP1, it binds two ligands and displays binding site cooperativity (174, 175). Binding of three BAs has also been shown (175), with ligands observed both within the interior cavity as well as on the surface of the protein (17, 175). FABP6 appears to show high selectivity for simultaneous binding of cholate and chenodeoxycholate (8, 175). Based on its BA binding properties and its expression in the distal small intestine where BAs are actively reabsorbed, it has long been thought that FABP6 plays a key role in intestinal lipid absorption by maintaining normal enterohepatic circulation of BAs. Indeed, mucosal to serosal transport of BAs, determined using everted gut sacs, was reduced in FABP6^−/−^ mice relative to WT controls (143). Physiological consequences, however, appear to be diet dependent and gender specific. Elevated fecal BA excretion relative to WT mice was found only in female FABP null mice fed a standard chow diet, in both males and females on a low-fat diet, and in both males and females fed a Western diet (62, 143). Increased fecal fat in FABP6 null mice relative to WT mice was found upon Western HFD feeding but not on a low-fat diet (62). Changes in the intestinal transcriptome and the gut microbiome of FABP6^−/−^ relative to WT mice may underlie these observations (23, 62).

It is now well appreciated that BAs function not only to promote the digestion, solubilization, and uptake of dietary fat but also as important metabolic regulatory molecules by serving as ligands for the nuclear hormone receptor FXR and the membrane-bound receptor TGR5 (25). FABP6 may thus play a role in the regulation of lipid and glucose metabolism (42). The apparent selectivity of FABP6 for binding specific low-abundance BA species provides indirect support for this notion (8), as does the greater fat mass of male FABP6^−/−^ mice despite their higher excretion of BA and fat (62). A potential signaling role for FABP6 is further suggested by studies in bladder cancer and human glioblastoma cells, where knockdown of FABP6 led to reduced cell growth and tumor invasiveness accompanied by reduced cell motility and migration and by cell cycle arrest (105, 137). It is worth noting that FABP6 is not highly expressed in normal bladder or neuronal cells; thus, it is not clear what if any role it may play in these cell types under nonpathological conditions.

Finally, an uncommon Thr79-to-Met FABP6 variant has been associated with a reduced risk of T2D in obese humans (43). While the mechanism underlying this relationship is not known, it points to a potential role for FABP6, expressed in small intestine, in regulating distal tissues and impacting whole-body energy metabolism.

## FABP7

FABP7 is expressed in brain, where it is specifically enriched in glial cells including astrocytes and microglia. Its function is thought to be related to its binding and trafficking of polyunsaturated fatty acids such as DHA, which is found abundantly in the central nervous system.

Hippocampal neurogenesis was shown to be reduced in FABP7 null mice, supporting a role in normal neuronal development (84). FABP7 knockdown increases permeability of the BBB, and exogenous administration of FABP7 following traumatic brain injury ameliorated BBB injury and restored impaired neurological function, suggesting that FABP7 is involved in the maintenance of barrier function (147). The disruption of sleep patterns upon FABP7 knockout or with a missense mutation supports a role in normal sleep function in several species including humans (55). FABP7 KO mice and mice treated with an FABP7 inhibitor were also found to have reduced nociception, indicating a role in pain perception which may be related to its endocannabinoid binding properties (10, 78, 79). The molecular mechanisms underlying these various proposed functions, however, remain largely unknown.

In addition to research on these potential physiological roles of FABP7, it has been studied in a pathological context as well. An association between FABP7 and glioblastoma has been known for some time, with a noteworthy inverse correlation between patient survival time and tumor levels of FABP7 (104). Indeed, overexpression of FABP7 was shown to increase the proliferation rate of the U87 glioblastoma cell line and of a xenograft model transplanted with these cells. By contrast, expression of a nonbinding mutant did not increase proliferation in these models, indicating that ligand binding is necessary for the FABP7 effects (81). Effects on proliferation were reported to be mediated via the p53 tumor suppressor and ERK1/2 signaling (172). FABP7 expression is also associated with increased cell migration and tumor invasiveness (31). Uptake of the ω3 PUFA DHA into glioblastoma patient-derived neural stem-like cells was diminished upon FABP7 knockdown, and the FABP7-dependent elevation in the ratio of DHA to the ω6 PUFA arachidonic acid (AA) was associated with a decrease in cell migration rate (27). The mechanisms underlying FABP7 effects on glioblastoma cell proliferation and migration are incompletely understood, but several intriguing possibilities have been proposed. Increased expression of FABP7 was associated with increased plasma membrane lipid order, and super-resolution microscopy showed the protein associated at the membrane surface in a punctate manner. These FABP7 domains were enriched at sites of cell migration and, interestingly, incorporation of DHA disrupted the FABP7-membrane associations and abrogated the FABP7-associated stimulation of cell migration (192). FABP7 is also reported to interact with αSyn. Treatment of U251 human glioblastoma cells with AA promoted αSyn aggregation in an FABP7-dependent manner, and a high affinity FABP7 synthetic ligand disrupted the FABP7–αSyn interaction and reduced αSyn oligomerization, thereby rescuing glial cells from cell death (20). FABP7 effects may also occur indirectly via regulation of lipid rafts, as it has also been reported to interact with ATP-citrate lyase, leading to acetylation of the caveolin-1 gene (82).

FABP7 has also been associated with a number of cancers outside the brain, notably breast cancer. Elevated levels of FABP7 have been reported in triple-negative breast cancer tissue (108), and studies in cell culture models have suggested that its overexpression leads to lower glucose utilization, cell cycle arrest, and cell death, possibly via increased generation of an oxidation product of linoleic acid, 13(S)-HODE (94, 95). FABP7 expression in brain was also found to be positively correlated with breast cancer brain metastases (28). An association between FABP7 expression in colon cancer tissues and cell proliferation as well as tumor growth has been observed, with effects dependent on MEK/ERK signaling (117). FABP7 has also been shown to be highly expressed in NSCLC patient tumor cells, and studies in NSCLC cell lines indicate that FABP7 exerts prometastatic effects via impaired β-catenin degradation leading to Wnt hyperactivation (9). FABP7 knockdown in melanoma cell lines decreased cell proliferation and invasiveness (160), and similar effects of FABP7 on Wnt/β-catenin signaling were recently reported (176). Ectopic expression of FABP7 has also been described in a subgroup of patients with diffuse large B cell lymphoma, in which a chimeric transcript of FABP7 with a transposable element promoter is expressed and is associated with DLBCL cell line proliferation (112).

Elevated levels of FABP7 in astrocytes have also been found in a mouse model of amyotrophic lateral sclerosis and were linked to increased inflammation via NF-κB signaling and neuronal toxicity in astrocyte-neuron cocultures (87). Similarly, in a mouse model of the autoimmune disease multiple sclerosis, an FABP7/FABP5 inhibitor was shown to reduce oxidative stress and inflammatory markers (19). FABP7 is also proposed to functionally interact with sortilin, the neuronal receptor for apolipoprotein E (apoE), with apoE3 leading to expression of FABP7 but the apoE4 variant, a major risk factor for Alzheimer’s disease, being associated with reduced FABP7 levels (6).

Clearly, many pathological states find elevated FABP7 levels to be detrimental; however, a limited number of reports indicate positive effects of FABP7 overexpression. In a glioma cell line, overexpression of FABP7 led to protection against reactive oxygen species (ROS) stress via increased accumulation of lipid droplets (LDs), whereas knockdown of FABP7 in astrocytes resulted in lower LD formation and increased ROS-mediated apoptosis; a reduction in apoptotic cell death upon FABP7 overexpression was also noted (74). In human skin cell carcinomas, FABP7 levels were reduced relative to control patient samples, and overexpression of FABP7 in cutaneous squamous cell carcinoma cell lines inhibited proliferation, invasiveness, and migration, with effects shown to be dependent on Notch signaling (168).

Overall, the role of bound ligand is likely critical to many if not all FABP7-dependent effects, with the specific ligand of critical importance in determining functional outcomes. Using malignant glioma cell lines, it was shown that DHA promoted FABP7 localization to the nucleus, resulting in a PPARγ-dependent reduction in cell migration. In contrast, AA promoted FABP7-dependent cell migration via reduction in PPARγ and increased cyclooxygenase-2 levels (35, 124). FA-specific roles for FABP7 have been proposed to mediate effects in Alzheimer’s disease, with AA binding stimulating inflammatory pathways and DHA imparting neuroprotection (129).

## FABP8

FABP8, also designated as peripheral myelin protein 2 (PMP2 or P2), is expressed by Schwann cells in the vertebrate peripheral nervous system (PNS) and in astrocytes in the human central nervous system. FABP8 is a stably attached peripheral membrane protein, a unique feature in the FABP family, and is located on the cytoplasmic side of compact myelin membranes. It is proposed to promote stacking of lipid bilayers via protein-membrane interactions at two membrane binding sites on opposite sides of the protein, rendering a crystal-like lattice structure (148).

The demyelinating type of Charcot-Marie-Tooth disease, a hereditary peripheral neuropathy, is caused mainly by defects in peripheral myelin proteins, including FABP8. Several missense mutations in FABP8 have been detected in patients with the disease (54). The mutations localize to a region situated at the bottom of the beta barrel, suggesting a mutation hot spot important for FABP8 structure or function. In vitro structure-function analysis of the mutant proteins has shown a decreased protein thermal stability but no change in membrane stacking capability compared with that of the WT protein (177). Transgenic mice expressing one of these FABP8 mutations, I43N, exhibit a significant reduction of motor nerve conduction velocity (68). It has been shown that FABP8 is able to control the transbilayer movement of plasma membrane sphingomyelin in a PI(4,5)P2-dependent manner and that I43N has an increased affinity for PI(4,5)P2, which could contribute to myelination defects in Schwann cells (2). It has also been shown that Schwann cells expressing FABP8 preferentially ensheathe motor axons and that the number of these Schwann cells is reduced in human amyotrophic lateral sclerosis (197).

Studies of FABP8 KO mice showed a mild effect on PNS function and an abnormal lipid composition during the period of most active myelination; perhaps surprisingly, no major structural changes in the myelin sheath were observed (198). A role for FABP8 in remyelination of peripheral nerves after nerve injury has also been demonstrated (164).

FABP8 expression was found to be upregulated in a SOX10-dependent manner in melanoma cells and to increase melanoma cell invasion (59).

## FABP9

FABP9 (TFABP, or PERF15) is expressed in mammalian adult testis, restricted to germ cells during spermiogenesis; it is found to increase with cell differentiation (136). In murine sperm, FABP9 is the major protein present in the perforatorium and associated perinuclear theca (145). FABP9 undergoes posttranslational phosphorylation and modification of thiol status, which are associated with sperm capacitation and stabilization of the flagellum, respectively (73). Mice lacking FABP9 remained fertile but showed increased sperm head abnormalities. The total lipid profile of the FABP9^−/−^ mice did not change when compared with that of WT controls; however, a compensation with FABP12 is not excluded (151).

Increased levels of FABP9 in PC human tissues detected by immunohistochemistry may have prognostic significance, and FABP9 depletion in PC cell lines resulted in reduced cell invasive potential in a PPARγ-independent manner (3).

## FABP12

The *FABP12* gene is located within a cluster that contains four other *FABP* genes (*FABP4*, *FABP5*, *FABP8*, and *FABP9*). It is expressed in retina together with FABP5 and FABP7, and in testis together with FABP9 (109).

In an analysis of the hypolipidemic effects of a traditional herb, *Alisma orientale*, liver transcriptome analysis in a diabetic rat model showed FABP12 to be upregulated, suggesting its participation in the reduction of blood lipid levels in this model (191).

The gene cluster that contains FABP4, FABP5, FABP8, FABP9, and FABP12 has been shown to be preferentially amplified and overexpressed in metastatic PC. Accordingly, the mRNA levels of all these proteins except FABP5 correlated with histological parameters and PC recurrence (107). In line with this, it was demonstrated that FABP12 promotes properties associated with cancer progression and lipid bioenergetic modifications, at least in part, through a PPARg-dependent pathway (106).

## CONCLUDING REMARKS

The mammalian FA-binding protein family members continue to be actively investigated, particularly insofar as their potential roles in the progression and perhaps etiology of numerous pathologies. The absence of apparent common functions and mechanisms of action for the different FABPs is intriguing, and indeed the FABP family is alone among small lipid-binding proteins in exhibiting a large degree of evolutionary diversity and tissue-specific expression patterns; by comparison, the genes encoding small cytosolic binding proteins for other lipids such as sterols, PLs, and sphingolipids are few and are expressed in all tissues. Indeed, the FABP family should be functionally expanded to include the retinoid-binding small cytoplasmic proteins, which in fact are part of the same evolutionary gene family and have been found not only to bind retinoids but also to have overlapping ligand binding specificities with the traditional FABP family (101). While FABP1 remains the family member with the widest reported range of ligands to date, it is clear that many and likely all of these proteins, which have large cavities that could theoretically accommodate a range of small lipid molecules, will be found to bind additional ligands. This has been clearly demonstrated for endocannabinoid binding to numerous FABPs, and a systematic assessment of the lipid ligands that bind to all the FABPs will provide additional important information about their functions.

**Figure 2:**
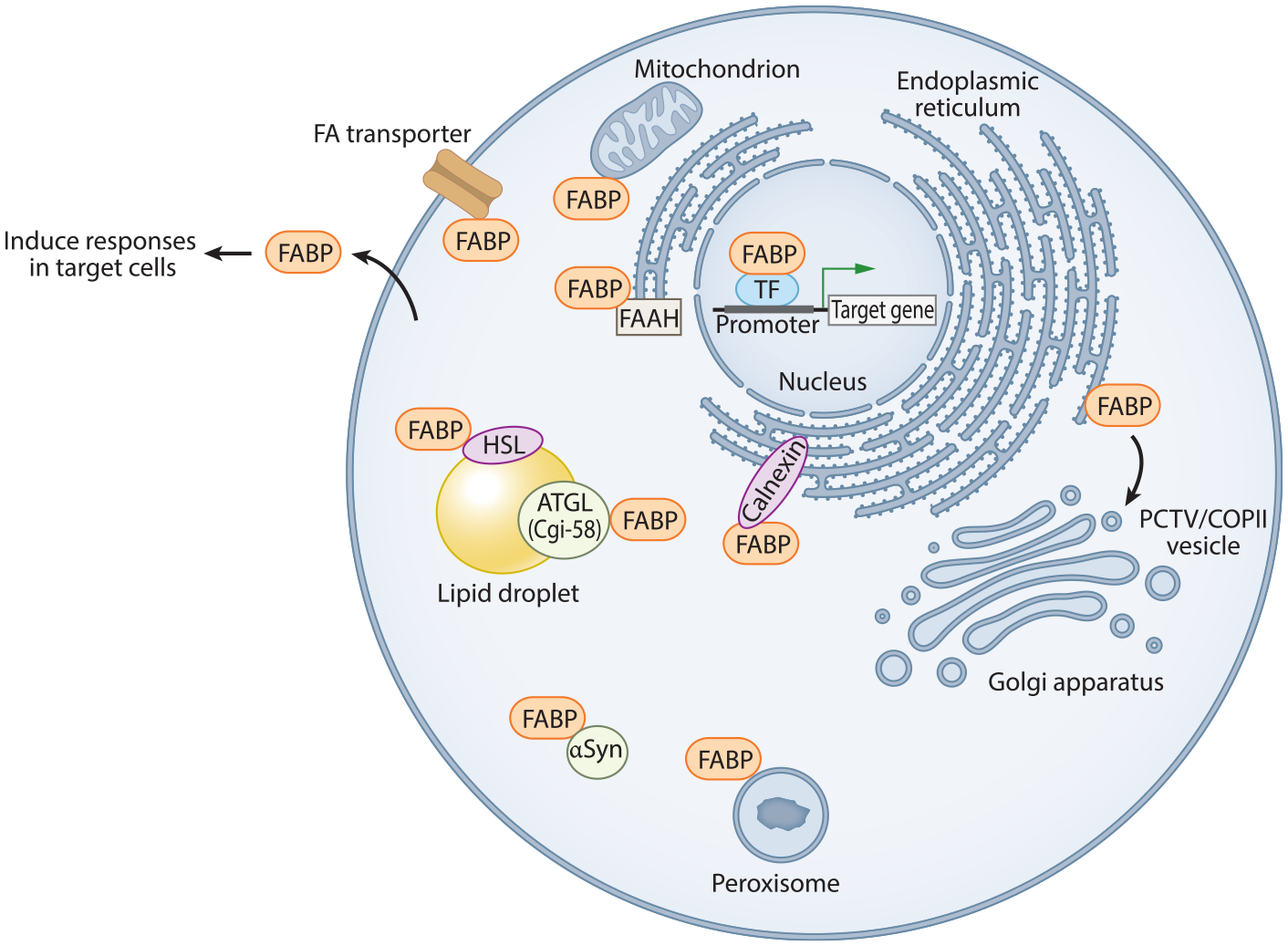
The multifunctional family of mammalian fatty acid (FA)-binding proteins. As described in the text, different FABPs are found to have specific functions within cells via transport of FA or other ligands to various subcellular destinations and via interactions with specific proteins. They are also reported to function in the circulation following noncanonical secretion from the cell. Not all proposed interactions are indicated, e.g., the stable peripheral membrane association of FABP8 in neuronal myelin sheath. Abbreviations: αSyn, α-synuclein; ATGL, adipose triacylglycerol lipase; Cgi-58, comparative gene identification-58; COPII, coat protein complex II; FA, fatty acid; FAAH, fatty acid amide hydrolase; FABP, fatty acid–binding protein; HSL, hormone-sensitive lipase; PCTV, prechylomicron transport vesicle; TF, transcription factor.

Within their cells of expression, the FABPs have been shown to participate in ligand trafficking with lipid metabolic enzymes or membrane transporters, for example, in the ER, as well as trafficking to the nucleus with subsequent effects on gene transcription. While it is likely that at one level these highly abundant proteins are acting to sequester high concentrations of FAs that could be deleterious to membranes, they are also very clearly playing regulatory roles within their cells. The specificity of FABP effects is likely secondary to interactions with target proteins, protein complexes, and/or target membrane domains. Several such interactions have been shown; among the interacting proteins for different FABPs are the PPAR transcription factors, lipolytic enzymes or their cofactors including the anandamide hydrolytic enzyme FAAH, membrane transporters such as CD36, and lipoprotein biogenesis protein complexes (Figure 2). A crucial step toward a comprehensive understanding of FABP function will include identification of the complete protein interactome for the various FABP family members.

**Figure 3:**
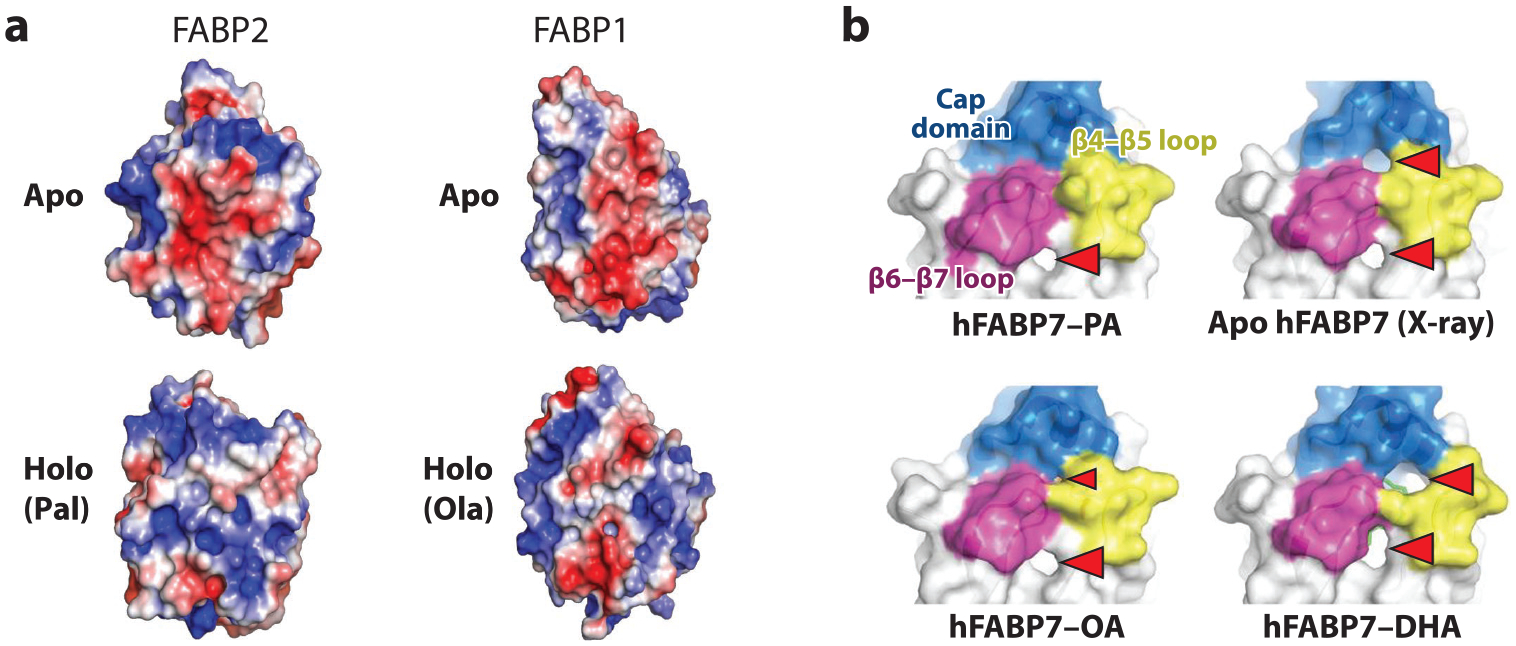
Ligand binding alters the surface conformations of the fatty acid–binding proteins (FABPs). (*a*) Comparative PyMOL visualization of electrostatic potential surfaces from nuclear magnetic resonance–based solution structures of rat FABP2 (1AEL, 1URE) and FABP1 (2JU3, 2JU8) in the absence (apo) and presence (holo) of bound ligands [palmitate (pal) or oleate (ola)], oriented with the α-helical portals at the top and the β-sheet barrels at the bottom. Blue and red colors designate positive and negative potential, respectively; a structure-based software plugin was used to align each apo-holo pair, so that variations in the surface shapes reflect true differences in the respective protein folds. Panel adapted with permission from Ruth E. Stark and Irving Rosario, The City College of New York. (*b*) Surface representations of the crystal structures of unliganded human FABP7 (6L9) or hFABP7 bound to palmitate (PA; 7E25), oleate (OA; 1FE3), or docosahexaenoate (DHA; 1FDQ). Differential holes in the surface are indicated by red triangles. Panel adapted from Reference 128 with permission from the International Union of Crystallography (https://doi.org/10.1107/S2059798321005763).

Given the multiple ligands that may bind to an FABP and the numerous subcellular fates that await these ligands, it is interesting to consider the mechanisms by which the FABPs might dictate a wide variety of functional outcomes, accounting for effects of specific ligands. Although structural analysis of the FABP family has focused largely on the binding cavity, examination of the surface characteristics of the proteins provides insight into how the binding or dissociation of ligands could dictate a specific downstream interaction. Figure 3a shows the electrostatic surfaces of apoFABP1 and apoFABP2, and of both proteins with bound FAs. While the FA is entirely contained within the binding cavity of the proteins, the effect of the bound ligand is seen to propagate to the protein surface, with marked differences in surface charge distribution between the apoand holoproteins. This could readily translate into specific structure-based interactions based on whether the FABP has bound ligand or not. Of interest in this regard is a recent analysis of the surface of FABP7 in the presence of different ligands (128). As shown in Figure 3b, differences in the surface structure in the interfacial region between the helical cap and the beta-barrel domains are clearly found between the unliganded protein and the protein with bound oleate, palmitate, or docosahexaenoate. Several functional outcomes of such ligand-induced structural changes have already been reported for the FABPs. For FABP4, PPARg-activating ligands stabilize the conformation of a noncanonical nuclear localization signal (NLS) in the protein, which delivers the complex and activates PPARγ in the nucleus, while for FABP5, it is PPARβ/δ agonists that stabilize the NLS, to promote PPARβ/δ activation (5, 57). In the case of FABP1, a change in the conformation of residues on the portal loops upon ligand binding modulates the electrostatic surface to promote its association with PPARα (142).

Various members of the FABP family have long been shown to be present in the circulation, and the use of specific FABPs as biomarkers for underlying tissue damage has been studied at length (187). While none of the FABPs have known secretory signal sequences, an exciting possibility, thus far most actively studied for FABP4 as discussed above, is that the FABPs may be secreted from their tissues of expression in a regulated manner and function either in the circulation itself, or via effects on downstream tissues. The fact that FABP null mice often have dramatic changes in whole-body energy metabolism would support the notion of FABPs acting as circulating regulatory factors; however, it is also possible that they may affect the secretion of hormones or other circulating messengers, within their tissues of origin. Clearly the mammalian FABPs remain an enigmatic and intriguing family of small proteins, with much remaining to be discovered.

## Figures and Tables

**Table 1:** Association of different FABPs with inflammatory and metabolic diseases

Disease	FABP(s)	Association	Reference(s)
Obesity	FABP1	Global FABP1 knockout enhanced HFD-induced obesity FABP1 level is increased in small intestine of obese humans	49, 196
	FABP4	FABP4 treatment induced insulin resistance in mice Incubation of hepatocytes with FABP4 induces insulin resistance, ER stress, and apoptosis	14, 92
Diabetes	FABP2	Ala54Thr associated with higher prevalence of T2D	64
	FABP4	High expression in retinal tissue in diabetic retinopathy	39
	FABP6	Thr79Met variant reduces risk of T2D in obese humans	43
NASH	FABP1	Global FABP1 knockout mice as well as hepatocyte-specific and intestine-specific FABP1 knockout attenuate NASH	130, 196
Kidney disease	FABP4	High expression in kidney tubular epithelia in kidney fibrosis	24
Heart failure	FABP3	FABP3 upregulated in MI mouse model	202
	FABP5	FABP5-deficient mice more susceptible to cardiac injury	50
Septic cardiomyopathy	FABP4	High levels of expression in septic heart muscle	167
Atherosclerosis	FABP4	High circulating FABP4 promotes vascular inflammation and plaque deposition	47, 99
		Macrophage-specific deletion of FABP4 protective against atherosclerosis	165
	FABP5	Macrophage-specific deletion of FABP5 protective against atherosclerosis	7
PD	FABP3	Elevated FABP3 in cerebrospinal fluid and serum in PD	102
		FABP3 necessary for aSyn uptake and aggregation in in vitro and in vivo PD models	45, 85, 156
ALS	FABP7	Elevated levels of FABP7 in astrocytes of ALS animal model	87
MS	FABP7 and FABP5	Inhibitor ameliorates injury in MS mouse models	19
Alzheimer’s disease	FABP7	Reduced FABP7 levels	6
Demyelinating Charcot-Marie-Tooth disease	FABP8	Missense mutations in *FABP8* gene	54

Abbreviations: αSyn, α-synuclein; ALS, amyotrophic lateral sclerosis; ER, endoplasmic reticulum; FABP, fatty acid–binding protein; HFD, high-fat diet; MI, myocardial infarction; MS, multiple sclerosis; NASH, nonalcoholic steatohepatitis; PD, Parkinson’s disease; T2D, type 2 diabetes.

**Table 2:** FABP dysregulation in cancer

Cancer type	FABP(s)	Association	Reference
Intestine	FABP1	FABP1 deletion protects against tumorigenesis in a small intestinal adenoma mouse model	32
		Enhanced FABP1 expression in small intestine epithelial cells promotes tumorigenesis	1
	FABP4	High expression has been shown in colon cancer tissue	173
	FABP5	FABP5 upregulation promotes proliferation in colon cancer cells	153
	FABP7	High expression correlates with cell proliferation in colon cancer tissues	117
Gastrointestinal stromal cancer	FABP3	High FABP3 protein expression	22
Gastric	FABP5	FABP5 knockdown decreases proliferation	201
Esophageal	FABP3	High FABP3 protein expression	29
Liver	FABP1	High FABP1 protein expression in hepatocellular carcinoma tissues	93
	FABP4	High expression in liver cancer stem cells	193
		High expression in hepatic stellate cells in hepatocellular carcinoma	26
Non-small cell lung cancer	FABP3	High FABP3 mRNA and protein expression in NSCLC tumor tissues	171
	FABP5	Knockdown decreases proliferation of lung adenocarcinoma cells	52
	FABP7	FABP7 is prometastatic in NSCLC cell lines	9
Breast	FABP2	Transcriptional modulation by hormones and microenvironment in culture cells	113
	FABP3	Transcriptional modulation by hormones and microenvironment in culture cells	113
		High protein expression in tumor tissues and adipose microenvironment	110
	FABP4	High protein expression in tumor tissues and adipose microenvironment	110
	FABP5	High protein expression in tumor tissues and adipose microenvironment	110
	FABP7	High expression of FABP7 in human triple-negative breast cancer tissue	108
Uterine sarcoma	FABP3	High FABP3 expression in tumor tissues	30
Ovary	FABP4	High expression in ovarian cancer tissues	56
Melanoma	FABP8	High FABP8 protein expression	59
	FABP7	FABP7 knockdown decreased proliferation in melanoma cell lines	176
Squamous cell carcinoma	FABP7	FABP7 levels reduced in patient samples	168
Renal	FABP5	FABP5 silencing inhibited proliferation of renal clear cell carcinoma cells	114
Prostate cancer	FABP5	Knockdown of FABP5 decreases proliferation in vitro	132
	FABP9	High FABP9 protein expression in cultured cells	3
	FABP12	FABP12 in metastatic prostate cancer	106
Bladder	FABP6	FABP6 knockdown decreases cell cycle progression and migration in vitro	105
Glioma	FABP4	High expression in glioblastoma tissue and cell lines	111
	FABP5	Suppression of FABP5 decreases proliferation in vitro	180
	FABP6	Knockdown of FABP6 in glioblastoma cells reduced tumor cell invasion	137
	FABP7	Overexpression of FABP7 increases proliferation of glioblastoma cells	81
		Overexpression of FABP7 protective in glioma cell line	74
Neuroblastoma	FABP4	High expression in tumor-associated macrophages in neuroblastoma	123
Leukemia	FABP4	High expression in acute myeloid leukemia cells	195
Lymphoma	FABP7	Ectopic expression in patients with large B cell lymphoma	112

Abbreviations: FABP, fatty acid–binding protein; NSCLC, non-small cell lung cancer.
